# Is exercise associated with health-related quality of life in children and adolescents with sickle cell disease using self- and parent proxy-reports?

**DOI:** 10.1007/s11136-026-04423-5

**Published:** 2026-11-07

**Authors:** Christina Constantinou, Nicola Payne, Olga van den Akker, Baba Inusa

**Affiliations:** 1 Psychology Department, Middlesex University, London, UK; 2 Department of AI and Personalised Health, Faculty of Life Sciences and Medicine, Kings College London, London, UK

**Keywords:** Children, Adolescents, Sickle cell disease, Health-related quality of life, Physical exercise

## Abstract

**Purpose:**

This study examined associations between demographics, disease severity indicators, levels of weekly mild, moderate and strenuous intensity exercise and self-reports and parental proxy-reports of health-related quality of life (HRQL) in children and adolescents with sickle cell disease (SCD). Concordance between parental and child/adolescent reports was also examined.

**Methods:**

This cross-sectional study adopted an interdependent-groups, correlational design. One-hundred and six children and ninety-six adolescents with SCD and one of their parents independently completed the Pediatric Quality of Life Inventory and the Godin-Leisure-Time Exercise Questionnaire. Parents also reported demographics and disease severity indicators.

**Results:**

Parents underestimated children’s/adolescents’ HRQL, although the effect was small, but reports of exercise were generally concordant. Fewer days missed from school and higher levels of moderate exercise were associated with better HRQL reported by children and their parent. Fewer days missed from school and fewer vaso-occlusive crises were associated with adolescent and parental reports of better HRQL. Higher levels of strenuous exercise were also associated with parental reports of better HRQL in adolescents.

**Conclusion:**

Disease severity indicators are associated with self-reports and parental reports of better child and adolescent HRQL but moderate intensity exercise also plays a role for children. Clinical practice should promote daily exercise and help parents understand that balance is needed between taking some precautions but not being overprotective so that exercise levels are insufficient and HRQL is impacted.

**Supplementary Information:**

The online version contains supplementary material available at https://doi.org/10.1007/s11136-026-04423-5.

## Abstract (plain language summary)

Sickle cell disease is a common blood disorder. Children and adolescents with sickle cell disease often report that they have a worse health-related quality of life than healthy children and adolescents. Factors that may contribute to this include episodes of severe pain and absence from school. They may also take part in less exercise compared to healthy peers, which may reduce their health-related quality of life. Exercise is important for general physical health and mental wellbeing. We carried out this study to better understand whether these factors are associated with children’s and adolescents’ health-related quality of life. We found that children with less absence from school and who took part in more moderate exercise reported a better health-related quality of life. Adolescents with less absence from school and fewer episodes of severe pain reported a better health-related quality of life. These results suggest that finding ways to most effectively manage pain and reduce school absence are crucial. Furthermore, parents should encourage children to engage in daily exercise.

**Supplementary Information:**

The online version contains supplementary material available at https://doi.org/10.1007/s11136-026-04423-5.

## Introduction

Sickle cell disease (SCD) is one of the most common genetic haemoglobinopathies [1]. The most prevalent and severe form is sickle cell anaemia (SCA) [2]. There are more than 14,000 people living with SCD in the UK [3]. SCD is most prevalent among individuals of Black African and Black Caribbean descent [3, 4], with over a third of children coming from single-parent families who are generally of lower socio-economic status (SES) [2].

In children and adolescents, the most prevalent complication is vaso-occlusive crises [5], where deformed rigid, sticky crescent shaped red blood cells become blocked in blood vessels [2]. Vaso-occlusive crises are characterised by the sudden onset of severe, acute painful episodes in multiple body sites which can last for hours to days [5, 6]. They may be triggered by physical or mental stress, temperature change or excessive strenuous exercise [5–7]. Other physical complications in children include anaemia, infection, acute chest syndrome, stroke and growth failure [6]. Children and adolescents with SCD may also suffer from psychosocial problems, such as, increased depression, low self-esteem, poor body image, fatigue and more social problems in comparison to their healthy peers, as well as hospitalisations and frequent absences from school [8–13]. The clinical manifestations and psychosocial consequences of SCD may impair health-related quality of life (HRQL) [14].

HRQL is a broad, multidimensional concept of well-being which gauges a person’s physical, social, emotional and school/work functioning [14]. The most prevalent measure of HRQL is the generic Pediatric Quality of Life Inventory (PedsQL™), which is available as child and adolescent self-report and parental proxy-report measures [14, 15]. Using this measure, parents generally report their child has a lower HRQL than child self-reports [16]. Furthermore, children and adolescents with SCD often report a lower HRQL compared to healthy children/adolescents [17–20], so it is important to understand the determinants of HRQL. Systematic reviews found that factors such as older age, being female and greater disease severity (e.g. more pain, vaso-occlusive crises, hospitalisations or school absences) predict lower self-reports of HRQL in children and adolescents with SCD [20, 21]. Additionally, lower reports of HRQL are associated with complications that are more prevalent with the onset of adolescence, for example, adolescents suffer from more vaso-occlusive crises compared to children [22]. Therefore, it may not be surprising that children report a higher HRQL than adolescents [23]. Consequently, the HRQL of children and adolescents should be examined separately.

Lifestyle behaviours may help in controlling symptoms of SCD, and so may have the potential to impact HRQL. For example, moderating participation in strenuous exercise may be helpful, as strenuous exercise induces metabolic and physiological changes that may have health consequences in SCD, such as triggering a vaso-occlusive crisis [7, 24, 25]. Although there is now evidence that short duration, high-intensity exercise may not result in adverse outcomes [26, 27], parents often deter exercise participation [28–30]. However, not participating in sufficient exercise may undermine HRQL. For example, in qualitative research, children and adolescents discussed that pain interfered with their daily lives including engaging in exercise [29, 31–33]. Furthermore, a study found that children with SCD feel their participation in exercise is limited, mainly by their parents and due to their condition, causing them to feel they are missing out [34].

It is recommended that healthy children and adolescents participate in 60 min of moderate (e.g. walking to school or cycling on level ground) to vigorous (e.g. running or football) intensity exercise every day [35]. However, fewer children and adolescents with SCD meet the daily recommendations compared to healthy children, assessed by both self-report questionnaires [36, 37] and accelerometers (a monitor worn by participants for seven consecutive days) [36]. Therefore, while children/adolescents with SCD may need to be careful when participating in exercise, and take precautions, such as resting regularly, remaining hydrated and staying warm [38, 39], exercise also has important health benefits [35]. Yet previous research on exercise in paediatric SCD rarely examines different intensities of exercise (e.g. mild, moderate and strenuous) and the association between exercise and HRQL has not been examined. Thus, the aim of the current study was to examine whether demographics, disease severity indicators, including number of vaso-occlusive crises and days missed from school, as well as levels of weekly mild, moderate and strenuous intensity exercise were associated with self- and parental proxy-reports of HRQL in children and adolescents with SCD. Since there are differences in HRQL reports between children and adolescents and between children/adolescents and parents, these groups were examined separately. Additionally, concordance between child/adolescent self-reports and parental proxy-reports of HRQL and exercise were also explored.

### Hypothesis 1

Indicators of less severe disease (fewer vaso-occlusive crises and days missed from school) and greater levels of exercise will be associated with child/adolescent self-reports and parental proxy-reports of better HRQL.

### Hypothesis 2

Parental proxy-reports of HRQL and exercise will be lower than child/adolescent self-reports.

## Methods

### Study design

The present cross-sectional study adopted an interdependent-groups, correlational design using questionnaires. Children and adolescents with SCD and one of their parents completed measures assessing the child/adolescent’s HRQL and exercise, so assessments of children and their parent and adolescents and their parent could be compared. The primary variable of interest was HRQL, and associations between this and demographics, disease severity indicators and exercise intensity scores were examined.

### Participants

A convenience sample included 106 children with SCD (aged 5 to 12 years) and one of their parents and 96 adolescents with SCD (aged 13 to 18 years) and one of their parents. Children and adolescents with SCD had to be attending the haematology outpatient clinic at one of three London hospitals, be aged 5 to 18 years of age, have no co-morbidities other than those associated with SCD, not be under-going inpatient treatment and be accompanied by a parent, as they were both required to complete the measures on the same day to reflect their current experience. The recruitment strategy is reported later in the Procedure. A sample size calculation using G*Power3 software suggested that a sample of at least 80 children and 80 adolescents and their parents would detect a 0.2 effect size with 80% power and a 5% significance level.

### Materials

#### Pediatric quality of life inventory (PedsQL™)

The generic PedsQL™ [40, 41] was used in the current study. This was chosen over the PedsQL™ SCD Module because it focuses on general functioning and overall HRQL, thus capturing broader aspects, rather than focusing specifically on SCD-related symptoms. Many children/adolescents with SCD engage in social activities, attend school regularly and manage sickle-related pain at home, and so are more likely to assess their experiences in relation to those of their healthy peers [42]. Use of the generic PedsQL™ also facilitates comparisons of scores with healthy children/adolescents or those with other chronic conditions if desired [21, 43].

It is available in parallel child self-report (for children aged 5 to 18 years) and parental proxy-report formats (for children aged 2 to 18 years) [40, 41]. It consists of 23 items assessing four dimensions of HRQL; physical functioning (eight items), emotional functioning (five items), social functioning (five items) and school functioning (five items). The questionnaire asks children and parents to think about how much of a problem the child has had with these areas in the past month. There is a 3-point Likert scale for children aged 5 to 7 years (e.g. 0 is ‘not at all’, 2 is ‘sometimes’ and 4 is ‘a lot’) and there is a 5-point Likert scale for all other child, adolescent and parent versions of the PedsQL™ (e.g. 0 is ‘never’, 1 is ‘almost never’, 2 is ‘sometimes’, 3 is ‘often’ and 4 is ‘almost always’).

HRQL scores are calculated by transforming responses on a scale which ranges from 0 to 100, where 0 is 100, 1 is 75, 2 is 50, 3 is 25 and 4 is 0, and computing a mean score, i.e. all of the items are summed and divided by 23 [40, 41]. HRQL scores range from 0 to 100, with a higher score indicating a higher/better HRQL [40, 41]. In the present study, child/adolescent self-reported and parental proxy-reported HRQL scores demonstrated good reliability (α = 0.90 to 0.93).

#### Godin-Leisure-time exercise questionnaire

The Godin-Leisure-Time Exercise Questionnaire [44–46] ascertains how many times in an average week children/adolescents engaged in strenuous (e.g. football, tennis, swimming fast), moderate (e.g. walking fast, easy swimming, riding your bike or scooter) and mild (e.g. easy walking, golf) intensity forms of exercise for at least 15 min. Participants were provided with examples of strenuous, moderate and mild exercise.

Each exercise intensity score is calculated by multiplying the number of times a week reported for each of strenuous, moderate and mild exercise by nine, five and three respectively, to create metabolic equivalents for each intensity category (i.e. a measure of exercise intensity based on oxygen consumption [44–46]). This questionnaire has been validated in children and adolescents, showing good test–retest reliability (α = 0.81) [47]. It has been used in adult and paediatric clinical populations with chronic conditions like cancer [48], and as a parental-reported measure, for example, completed by mothers of children with Fragile X Syndrome [49]. In the present study, child/adolescent self-reports and parental reports were taken. Scores for strenuous, moderate and mild intensity exercise are generally summed to create a total score. In the present study, similarly to previous research in, for example, adolescent cancer survivors [50], these were reported separately to retain information about each intensity of exercise.

#### Demographics and disease severity indicators

Parent reported demographics, including the child/adolescent’s gender, age, phenotype and ethnic group and the parent’s marital status, highest educational attainment level and questions that assessed the child’s disease severity, e.g. how many vaso-occlusive crises and how many days the child has missed from school in the last twelve months due to SCD. See Table 1.

### Procedure

Ethical approval was was obtained for this study from the authors’ university research ethics committee, the NHS Research Ethics Committee (reference number 17/LO/0322) and the Research and Development departments of each NHS hospital/Trust. The recruitment strategy consisted of hospital staff identifying 750 patients attending the sickle cell outpatient clinics at three London hospitals who met the eligibility criteria outlined earlier under Participants. A week prior to data collection commencing, they were posted age-appropriate information sheets to their homes. Following this, over an eight-week period, participants were approached during their outpatient clinical appointment and asked if they would like to participate. Thus, participants were volunteers and self-selection bias may be an issue. As patients had outpatient clinical appointments every six months, around 250 patients attended the clinic during the period of data collection. The first author read through the age-appropriate information sheets with eligible participants and the child/adolescent and parent provided consent for their own participation and parents provided consent for their child’s participation. All participants completed the generic PedsQL™ and Godin-Leisure-Time Exercise Questionnaire independently with the first author’s assistance if needed. Parents also completed questions about demographics and disease severity indicators. Interpreter support was available at the hospitals for those who did not understand English.

### Statistical methods

IBM SPSS Statistics for Windows, Version 25.0 (IBM Corp., Armonk, NY, USA) was used to analyse the data. There were no missing data. Statistical significance was set at *p* < .05. To test for differences and concordance between child-parent and adolescent-parent reports of HRQL and exercise (mild, moderate and strenuous) paired-samples t-tests and pearson’s correlation analyses were conducted. Four hierarchical multiple linear regression analyses were conducted to examine which variables were associated with HRQL reported by children, their parents, adolescents and their parents. Demographics were entered on the first step, disease severity indicators on the second step and exercise participation on the third step. Self-reports of exercise were included when predicting self-reports of HRQL and parental reports of exercise were included when predicting parental-proxy reports of HRQL. The demographics to be included in the first step were selected based on statistical significance in preliminary univariate analyses, which included independent samples t-tests, one-way ANOVAs and pearson’s correlation analyses to examine relationships between demographics and HRQL reported by children, their parents, adolescents and their parents.

## Results

Sample characteristics are described in Table 1. Table 1 demonstrates that most children (78%) and adolescents (80%) had the most severe form of SCD (i.e. HbSS), that both groups had on average two vaso-occlusive crises in the last year but that adolescents had missed on average four more days of school in the last year than children. The table also highlights male and female gendered children/adolescents, as identified by their parent, were equally represented in the sample, and no parent took the opportunity to identify their child/adolescent in any other way. However, most parents identified as female, since mothers were more likely to accompany children to their hospital appointment, and half were single.

**Table 1:** Demographic characteristics of children (*n* = 106) and adolescents (*n =* 96)

	Children M(SD) or %n	Adolescents M(SD) or %n
Age	8.75 (2.31)	15.29 (1.61)
Gender	51% Male	50% Male
Ethnicity	75% Black African, 21% Black Caribbean, 5% Mixed	76% Black African, 21% Black Caribbean, 3% Mixed
SCD phenotype	78% HbSS, 22% HbSC	80% HbSS, 20% HbSC
Vaso-occlusive crises	1.96(2.37)	1.95(2.23)
	31% no crises 25% 1 crisis 15% 2 crises 8% 3 crises 14% 4 crises 1% 5 crises or more	28% no crises 24% 1 crisis 22% 2 crises 6% 3 crises 9% 4 crises 9% 5 crises or more
Missed days from school	7.75(9.93)	12.17(22.80)
Parent’s age	40.39(6.19)	48.05(6.24)
Parent’s gender	19% Male	17% Male
Parent’s marital status	48% Married/cohabiting, 52% Single/separated/divorced	48% Married/cohabiting, 52% Single/separated/divorced
Highest educational attainment	36% Higher degree or degree 22% Foundation degree or diploma 43% A-levels or below	43% Higher degree or degree, 24% Foundation degree or diploma 33% A-levels or below

*HbSS* sickle cell anaemia, *HbSC* haemoglobin sickle C disease, *A-level* Advanced level

Descriptive statistics for child/adolescent self-reported and parental proxy-reported HRQL and the different intensities of exercise are shown in Table 2. This table also shows the results of paired-samples t-tests examining differences between child and parent and adolescent and parent reports of HRQL and exercise.

**Table 2:** Comparison of mean parental reports and child/adolescent self-reports of HRQL and weekly exercise

	Child self-report	Parental proxy-report	t	d	Adolescent self-report	Parental proxy-report	t	d
	M(SD)	M(SD)			M(SD)	M(SD)		
HRQL	73.16 (16.40)	71.08 (17.16)	2.23*	0.22	73.05 (16.99)	71.68 (17.16)	2.31*	0.23
Exercise								
Strenuous	68.8 (46.64)	68.77 (51.04)	0.03	0.003	50.44 (48.06)	47.63 (48.53)	1.38	0.14
Moderate	33.16 (18.26)	34.95 (17.61)	− 1.16	− 0.11	27.50 (19.64)	27.08 (20.15)	0.34	0.03
Mild	32.35 (14.07)	32.38 (12.54)	− 0.03	− 0.003	30.28 (16.51)	27.91 (16.07)	2.24*	0.23

*p < .05. **p < .01. ***p < .001

Table 2 indicates that children and adolescents reported higher mean HRQL compared to parents, although this was a small effect and pearson’s correlations showed strong significant positive associations between child-parent (*r* = .84) and adolescent-parent (*r* = .94) reports of HRQL. Pearson’s correlations for the relationships between child-parent and adolescent-parent reports of exercise also revealed strong positive associations ranging from *r* = .61 to 0.92, and Table 2 shows only one significant mean difference; adolescents reported participating in more weekly mild intensity exercise compared to parental reports, although this was a small effect. 

Preliminary univariate analyses conducted to select demographics to include in the four hierarchical multiple linear regression analyses demonstrated that most variables in Table 1 were not related to HRQL. For the sake of brevity, statistics are only reported for statistically significant findings. Please see supplementary material for all univariate analyses. Independent-samples t-tests found that there was no statistically significant difference in HRQL between male and female children/adolescents (where their parent was asked to report their gender). One-way ANOVAs found no statistically significant difference in HRQL when comparing parents with different levels of educational attainment. However, one independent-samples t-test indicated there was a statistically significant difference in self-reports of HRQL between adolescents from married (*M* = 76.25, *SD* = 14.52) and single (*M* = 70.11, *SD* = 18.63) parent families, with the former reporting better HRQL (*t*(94) = -1.79, *p* = .038). Finally, there was a significant correlation between children’s age and parental proxy-reported HRQL (*r* = − .20, *p* < .05), with parents of older children reporting worse HRQL. Thus, the four hierarchical multiple linear regression analyses reported by children and their parents and adolescents and their parents included child’s age and parent’s marital status on step one, vaso-occlusive crises and days missed from school on step 2 and the three exercise intensity measures (mild, moderate and strenuous exercise) on the third step.

**Table 3:** Hierarchical multiple regression analyses examining variables associated with child HRQL

	Child self-reported HRQL			Parent proxy-reported HRQL		
	β from final step	*R*^2 ^change at each step	F change	β from final step	*R*^2 ^change at each step	F change
STEP 1		0.023	1.21		0.039	2.11
Child’s age	0.02			− 0.06		
Parents marital status	0.04			0.00		
STEP 2		0.264	18.74***		0.241	16.87***
Vaso-occlusive crises	− 0.05			− 0.10		
Days missed from school	− 0.40**			− 0.28*		
STEP 3		0.046	2.27		0.090	4.65**
Strenuous exercise	0.08			0.11		
Moderate exercise	0.20*			0.24**		
Mild exercise	0.00			0.09		

Betas only in the final model are reported

*p < .05. **p < .01. ***p < .001

F change degrees of freedom are (2,103) at step 1, (2,101) at step 2 and (2,99) at step 3

As shown in Table 3, the final model for child-reported HRQL explained 33% of the variance (*F*(7,98) = 7.02, *p* < .001). The final model for parental proxy-reported HRQL for children explained 37% (*F*(7,98) = 8.21, *p* < .001). In both models, fewer days missed from school and greater engagement in moderate exercise were statistically significantly associated with better HRQL.

**Table 4:** Hierarchical multiple regression analyses examining variables associated with adolescent HRQL

	Adolescent self-reported HRQL			Parent proxy-reported HRQL		
	β from final step	*R*^2 ^change at each step	F change	β from final step	*R*^2 ^change at each step	F change
STEP 1		0.070	3.49*		0.058	2.89
Adolescent’s age	− 0.08			− 0.06		
Parents marital status	0.07			0.07		
STEP 2		0.324	24.28***		0.299	21.18***
Vaso-occlusive crises	− 0.23*			− 0.22*		
Days missed from school	− 0.40***			− 0.32**		
STEP 3		0.027	1.35		0.085	4.50**
Strenuous exercise	0.17			0.24*		
Moderate exercise	− 0.03			0.03		
Mild exercise	0.02			0.10		

Betas only in the final model are reported

*p < .05. **p < .01. ***p < .001

F change degrees of freedom are (2,93) at step 1, (2,91) at step 2 and (2,89) at step 3

As shown in Table 4, the final model for adolescent-reported HRQL explained 42% of the variance (*F*(7,88) = 9.11, *p* < .001). The final model for parental proxy-reported HRQL for adolescents explained 44% (*F*(7,88) = 10.00, *p* < .001). In both models, fewer vaso-occlusive crises and fewer days missed from school were statistically significantly associated with better HRQL. Greater engagement in strenuous exercise was also associated with better parental proxy-reported HRQL only.

## Discussion

The findings partially support Hypothesis 1; fewer days missed from school and fewer vaso-occlusive crises were associated with better HRQL, although the latter relationship was only when reported by adolescents and their parents. Additionally, moderate exercise was associated with better HRQL reported by children and their parents, and increased strenuous exercise was associated with better HRQL reported by parents of adolescents only. Hypothesis 2 was also partially supported, as parents slightly underestimated children’s/adolescents’ HRQL. Reports of exercise were generally concordant, although parents slightly underestimated adolescents’ levels of mild exercise.

Before discussing these findings, we acknowledge that in a high-income country, such as the UK, patients with SCD have access to free healthcare and support services, as well as advances in medical treatments. A study comparing low/middle-income and high-income countries found that SCD patients from the former were less likely to receive a diagnosis by five years of age, were less aware of treatment options, and had reduced insurance coverage for costs [51]. Moreover, a review concluded that lower SES was associated with poorer HRQL in SCD, through reduced access to routine healthcare, greater reliance on emergency services, higher financial burden, and missed clinical appointments, within high and low-income countries [21, 52]. Thus, the factors that shape HRQL in paediatric HRQL, and the findings of this study, may not be generalisable to all countries.

Nevertheless, previous SCD research supports that parents underestimate their child’s/adolescents’ HRQL [16, 18, 53, 54]. It may be particularly difficult for parents to gauge less directly observable domains of HRQL, such as emotional well-being, compared to the physical domain [15, 53], especially in adolescents who may not want to discuss emotional issues and are likely to spend a lot of time outside the family home [55]. However, mean parental proxy-reports were only slightly lower than child/adolescent self-reports, and the effect was small, so parents are not that inaccurate, and parent and child/adolescent reports of HRQL were significantly correlated. Discordance between child/adolescent and parental proxy-reports may be more pronounced in certain groups. For example, greater discordance has been found where children/adolescents have less active SCD symptoms, and where parents report greater levels of depression [55]. These factors were not examined in the present study, but future research could investigate this further.

In the current study parents underestimated adolescents’ levels of mild exercise, although the effect was small. It may be that adolescents have greater freedom in their leisure time than children [55] and therefore parents are not fully aware of adolescents’ participation in mild forms of daily exercise such as walking. It is also possible that parents are not fully aware of their moderate and strenuous exercise, as adolescents may not disclose some activities due to fear of parental disapproval. However, there was agreement in child/adolescent and parental reports of these forms of exercise. This may also support the reliability of the method used to assess exercise. Much past research on paediatric SCD has also used self-report measures of exercise [25, 36, 37], which may not be reliable due to recall bias and social desirability bias [56]. However, research has shown that objective measures (an accelerometer) and self-reports yielded the same results in children/adolescents with SCD [36].

The main aim of the present study was to examine variables associated with HRQL. The findings demonstrated that disease severity indicators were associated. Fewer days missed from school was associated with better child/adolescent self-reported and parental proxy-reported HRQL, which supports previous research [57, 58]. Past evidence has consistently shown that more frequent hospital admissions, which would mean that children are likely to be absent from school, is related to worse HRQL [18, 58, 59]. Frequent short and long-term school absenteeism in children/adolescents with SCD [11] may lead to poorer academic attainment compared to their peers [60] and affect social relationships and psychological well-being.

In the current study, fewer vaso-occlusive crises were also associated with better adolescent HRQL (both self- and parental proxy-reports). Past research has found that more vaso-occlusive crises are linked to worse HRQL in both children and adolescents with SCD [53, 61–64]. The fact that this finding was only obtained in adolescents in the present study may be explained by the greater number of vaso-occlusive crises experienced by adolescents compared to children according to parental reports (9% vs. 1%, respectively, experienced five or more crises in the last 12 months). The frequency of vaso-occlusive crises tends to increase during adolescence [22].

The present study also found that greater moderate exercise was associated with better child HRQL (both self- and parental proxy-reported), and greater strenuous exercise was associated with better adolescent HRQL (parent proxy-reported only). Exercise participation may not be an issue for adolescents’ HRQL as they may be preoccupied with other matters such as socialising and preparing for their school examinations [31, 65], so they may not be so concerned by any limits placed on strenuous exercise by parents. However, as greater strenuous exercise was related to parental proxy-reports of adolescents’ HRQL, it may be that parents believe that participation in strenuous exercise is important to adolescents. Parents may, therefore, have concerns about the impact of moderating this form of exercise in an effort to prevent triggering a vaso-occlusive crisis [25, 37].

For children, moderate exercise (not strenuous) was associated with better self- and parental proxy-reports of HRQL. Research shows physical activities in children with SCD are somewhat limited [32, 34] and they participate in significantly less sports at school and in their leisure time compared to their healthy peers [36]. While some of this may be strenuous exercise, the findings of a positive relationship between moderate exercise and HRQL suggest that it may be beneficial for HRQL to ensure that SCD does not undermine participation in moderate exercise. Participating in exercise has been reported by children with SCD to be important to them because they do not want to feel as though they are missing out, it provides them with opportunities for socialising and playing with their friends, and they do not want to be different to their friends [34]. Recent research provides evidence that even short duration high-intensity exercise may be safe in patients with SCD. A systematic review of studies of 8 to 12 min of Cardiopulmonary Exercise Testing (CPET) found this did not result in SCD-related complications [27]. Moreover, a study examining CPET and Controlled Intensity Interval Training of longer duration found both moderate and vigorous exercise were tolerated in 10 to 21 year old non-asthmatic patients with SCA, with no adverse outcomes [26].

While the current study suggests possible benefits of maintaining a sufficient level of participation in moderate exercise, it is acknowledged that this is a cross-sectional study, and that moderate exercise was only related to better HRQL reported by children and their parents but not by adolescents and their parents. Additionally, the proportion of variance in HRQL explained by all forms of exercise ranged from 3 to 9%. Earlier studies have not examined the association between exercise and HRQL in children/adolescents with SCD, but in adults with SCD, regular exercise was linked to improvements in some domains of HRQL, including general health, social function and vitality, and reduced pain [66]. This provides some support for moderate exercise also being linked to better HRQL in the present study. However, disease severity indicators played the most important role, explaining 24 to 32% of the variance in the HRQL of children and adolescents with SCD. Overall, the variance in HRQL explained by the regression models ranged from 33 to 44%. Therefore, a proportion of the variance in HRQL was attributable to factors not measured in the present study.

### Implications

The disparity between child/adolescent self-reports and parental proxy-reports of HRQL, while small, could be addressed, since parents make health and lifestyle decisions affecting their children. Health professionals could encourage communication between children/adolescents and parents to help parents gain greater insight into their child’s HRQL, and areas that need addressing. Understanding factors underlying discrepancies between child/adolescent and parental perceptions of HRQL may have implications for clinical decision-making, and this could be investigated by further research. The current study also found that disease severity indicators were associated with HRQL reports, so strategies to reduce the number of vaso-occlusive crises and days missed from school are important. Information should be shared between healthcare professionals, parents, children/adolescents and schools, including information about exercise, since moderate exercise was associated with HRQL in children and exercise is important for the general health of children/adolescents [35]. Health professionals should provide specific advice to parents at an early stage (i.e. when their child is a toddler), and later to schools, focusing on promoting exercise and highlighting the importance of daily moderate exercise (e.g. walking, cycling, playground activities) [35]. Moderate exercise is emphasised here due to the relationship found between this and HRQL in children, but evidence now suggests that participation in short duration, high-intensity exercise may be safe for children and adolescents with SCD. Further research is needed, particularly regarding participation in different forms of longer duration, high-intensity exercise, as well as team sports and contact sports, in order to develop guidelines. However, child and parental perceptions that vigorous exercise will trigger a vaso-occlusive crisis [e.g. 34] could be changed through education in health settings. CPET might be considered to examine the safety for individual child/adolescent patients [27]. To the extent that exercise is related to HRQL, it may even help as a strategy in coping with SCD [34]. A balance needs to be struck between taking some precautions when it comes to exercise but parents not being overprotective so that exercise levels are insufficient and HRQL is impacted.

### Limitations

There are some potential limitations to this study. Recruiting a convenience sample of volunteers during outpatient clinical appointments may mean that participants were more likely to be utilising support and adhering to medical advice, and thus may have a better HRQL than other children/adolescents with SCD. They may also have traits/characteristics that contribute to a better HRQL, such as higher SES, so self-selection bias may be an issue. Use of the Godin-Leisure-Time Exercise Questionnaire may also be a limitation as it has not often been used with children, and exercise intensities (i.e. mild, moderate and strenuous) in this measure are normally summed. Nevertheless, it has been used in children and adolescents [47] and exercise intensities have been reported separately [50]. Additionally, while social desirability bias may be an issue, subjective and objective measures of exercise have been found to be consistent in this population [36]. Also, the present study obtained reports of exercise from two sources; children/adolescents and parents and these reports were generally consistent. This study also obtained HRQL reports from two sources, but the disease severity indicators were only obtained from parents. Child/adolescent reports may be valuable in future research, as well as other, possibly objective, measures of disease severity. However, some research has found no differences in associations with HRQL in children with SCD when using hospital records or parental reports of disease severity [57]. This study used the generic PedsQL™ rather than the PedsQL™ SCD Module, due to a desire to focus on overall HRQL rather than SCD-related symptoms. However, it is acknowledged that the PedsQL™ SCD Module may improve measurement specificity when assessing HRQL, thereby enabling more sensitive discrimination among children/adolescents with SCD [21]. Finally, the study had a cross-sectional design, so cannot show whether greater participation in moderate exercise leads to better HRQL in children. It is also possible that better HRQL facilitates greater participation in exercise. Thus, a longitudinal study is recommended, and this could also look at the impact of other lifestyle factors such as hydration or risky behaviours such as alcohol or drug use.

## Conclusion

This study highlights the negative relationship between disease severity and HRQL of children and adolescents with SCD and suggests that exercise may also play a role. Parents, healthcare professionals and schools should work together to try to reduce vaso-occlusive crises or absences from school due to SCD. While strenuous exercise was linked to parental proxy-reports of adolescents’ HRQL, it was not related to adolescents’ self-reports, but moderate exercise was linked to children’s HRQL. Education about exercise may be valuable. A balance should be struck to avoid unintentional negative implications for HRQL if parents are overprotective when it comes to exercise, especially now evidence on the safety of exercise in SCD is changing.

## Supplementary Information

Below is the link to the electronic supplementary material.

Supplementary Material 1

## Data Availability

No datasets were generated or analysed during the current study.
