# Supplementary material for: Is exercise associated with health-related quality of life in children and adolescents with sickle cell disease using self- and parent proxy-reports?
Source: Qual Life Res. 2026 Nov 7;35(12):325. doi: 10.1007/s11136-026-04423-5 (PMC13744541; doi:10.1007/s11136-026-04423-5)
Supplement: Supplementary file 1 [file 11136_2026_4423_MOESM1_ESM.docx]

**Supplementary Material**

**Table S1** Child/adolescent gender differences in mean child and adolescent self-reports and parental-proxy reports of HRQL

|  | Self-reported HRQL  Male Female  M(SD) M(SD) | *t* | Parental proxy-reported HRQL  Male Female  M(SD) M(SD) | *t* |
| --- | --- | --- | --- | --- |
| Child HRQL | 71.42(17.94) 74.98(14.58) | -1.12 | 68.72(17.29) 73.54(16.83) | -1.45 |
| Adolescent  HRQL | 75.34(15.57) 70.77(18.17) | 1.34 | 74.66(15.73) 68.70(18.15) | 1.72 |

*Note.* * *p* < .05*.* ** *p* < .01*.* *** *p <* .001.

**Table S2** Correlations between child/adolescent age and mean child and adolescent self-reports and parental-proxy reports of HRQL

|  | Child self-reported HRQL | Parental proxy-reported HRQL | Adolescent self-reported HRQL | Parental proxy-reported HRQL |
| --- | --- | --- | --- | --- |
| HRQL | -.14 | **-.20*** | -.16 | -.15 |

*Note.* * *p* < .05*.* ** *p* < .01*.* *** *p <* .001.

**Table S3** Parental marital status differences in child and adolescent self-reports and parental-proxy reports of HRQL

|  | Self-reported HRQL  Single Married  M(SD) M(SD) | *t* | Parental proxy-reported HRQL  Single Married  M(SD) M(SD) | *t* |
| --- | --- | --- | --- | --- |
| Child HRQL | 72.06(17.09) 74.36(15.69) | -.72 | 70.34(18.39) 71.89(15.87) | -.46 |
| Adolescent HRQL | 70.11(18.63) 76.25(14.52) | **-1.79*** | 69.02(17.80) 74.57(16.12) | -1.60 |

*Note.* * *p* < .05*.* ** *p* < .01*.* *** *p <* .001.

**Table S4** Parental educational attainment differences in child and adolescent self-reports and parental-proxy reports of HRQL

|  | Self-reported HRQL  Group 1 Group 2 Group 3  M(SD) M(SD) M(SD) | *F* | Parental proxy-reported HRQL  Group 1 Group 2 Group 3  M(SD) M(SD) M(SD) | *F* |
| --- | --- | --- | --- | --- |
| Child  HRQL | 72.40(17.12) 72.02(15.00) 74.40(16.73) | 0.22 | 71.28(15.99) 67.34(16.05) 72.83(18.65) | 0.78 |
| Adolescent HRQL | 74.08(15.92) 70.98(17.69) 73.27(18.05) | 0.24 | 71.40(17.36) 70.89(15.38) 72.54(18.47) | 0.07 |

*Notes.* * *p* < .05*.* ** *p* < .01*.* *** *p <* .001.

*Group 1* = *higher degree or degree. Group 2 = foundation degree or diploma. Group 3 =* *Advanced levels or below.*
